# Single-Cell Transcriptional Heterogeneity of Neutrophils During Acute Pulmonary *Cryptococcus neoformans* Infection

**DOI:** 10.3389/fimmu.2021.670574

**Published:** 2021-04-29

**Authors:** M. Elizabeth Deerhake, Estefany Y. Reyes, Shengjie Xu-Vanpala, Mari L. Shinohara

**Affiliations:** ^1^ Department of Immunology, Duke University School of Medicine, Durham, NC, United States; ^2^ Department of Molecular Genetics and Microbiology, Duke University School of Medicine, Durham, NC, United States

**Keywords:** neutrophils, fungal infection, pulmonary, *Cryptococcus neoformans*, heterogeneity, single-cell RNA sequencing (scRNA-seq)

## Abstract

Neutrophils are critical as the first-line defense against fungal pathogens. Yet, previous studies indicate that neutrophil function is complex during *Cryptococcus neoformans (Cn)* infection. To better understand the role of neutrophils in acute pulmonary cryptococcosis, we analyzed neutrophil heterogeneity by single-cell transcriptional analysis of immune cells in the lung of *Cn*-infected mice from a published dataset. We identified neutrophils by reference-based annotation and identified two distinct neutrophil subsets generated during acute *Cn* infection: A subset with an oxidative stress signature (Ox-PMN) and another with enhanced cytokine gene expression (Cyt-PMN). Based on gene regulatory network and ligand-receptor analysis, we hypothesize that Ox-PMNs interact with the fungus and generate ROS, while Cyt-PMNs are longer-lived neutrophils that indirectly respond to *Cn*-derived ligands and cytokines to modulate cell-cell communication with dendritic cells and alveolar macrophages. Based on the data, we hypothesized that, during *in vivo* fungal infection, there is a division of labor in which each activated neutrophil becomes either Ox-PMN or Cyt-PMN.

## Introduction

Opportunistic fungal infections are a serious complication of immunosuppression in patients undergoing transplantation, patients with HIV-AIDS, and those with immunosuppression induced by leukemia or lymphoma (1). Among opportunistic fungal infections, *Cryptococcus neoformans* (*Cn*) is one of the pathogens with the highest disease burden and risk of complications. Inhaled from the environment, *Cn* begins as a primary pulmonary infectious agent and can disseminate through the vasculature to the central nervous system (CNS) resulting in meningoencephalitis (1).

Neutrophils are critical as the first-line of defense against fungal pathogens, effectively engulfing and killing *Cn*, arguably more efficacious than monocytes (2, 3). For instance, neutrophils produce the majority of reactive oxygen species (ROS) during cryptococcosis in attempts to control and clear the infection (4, 5). Treatment with granulocyte colony-stimulating factor (G-CSF) decreased fungal burdens in mice with cryptococcosis (6) and reduced risk of infection in AIDS patients (7), suggesting that neutrophils contribute to host immune defenses during cryptococcal infection. Using *in vivo* antibody-mediated neutrophil depletion, studies have demonstrated that neutrophils are crucial for the clearance of intravascular *Cn* in the lung and brain (8, 9). Additionally, myeloperoxidase (MPO), the neutrophil azurophilic granule factor, is protective in murine *Cn* infection when administered *via* intravenous (*i.v*.) and intranasal (*i.n.*) routes (10).

However, the role of neutrophils in *Cn* infection is not straightforward. In contrast to intravascular infection, neutrophil depletion leads to a paradoxical increase in survival in the setting of intratracheal infection (11–13). One explanation is that acute neutrophil recruitment to the lung and the associated anti-fungal response following *Cn* infection could cause host-detrimental inflammation, leading to tissue damage. However, neutrophil depletion also leads to an increase – not a decrease – in inflammatory cytokine production in the lung (11, 14); and increased inflammatory cytokine levels may promote anti-fungal immunity and increase host survival. Thus, neutrophils may be detrimental both through off-target tissue damage or by reducing immune responses.

The *in vivo* functions of neutrophils in pulmonary *Cn* infection are not well understood and likely involve a complex balance of antifungal and regulatory activities. In addition, neutrophils have a short half-life, and *ex vivo* analyses are extremely challenging. To map neutrophil heterogeneity during acute pulmonary infection with *Cn*, we analyzed a single-cell RNA sequencing (scRNA-seq) dataset of lung extra-vascular and intra-vascular immune cells. Following reference-based annotation of neutrophils, we identified multiple neutrophil subsets with distinct transcriptional profiles, including two subsets which were found in *Cn-*infected mice, not in naïve mice. Using both gene regulatory network and ligand-receptor analyses, we discovered predicted pathways which may contribute to neutrophil subset identity and modulate interactions between neutrophils and other myeloid cells during acute pulmonary *Cn* infection. These preliminary data lead us to further hypothesize the distinct functions and longevity of neutrophil subsets. Further characterization of these distinct neutrophil subsets may provide potential therapeutic targets to enhance anti-*Cn* immunity.

## Methods

### Dataset Availability

The single-cell RNA sequencing dataset analyzed in this study (NCBI GEO: GSE146233) was previously published (15) and focused on analysis of alveolar macrophages (AM) (15). This dataset has not previously been used to analyze neutrophils or other immune cell populations.

### Sample Preparation and Single-Cell RNA Sequencing

Samples were prepared as previously described (15): Mice heterozygous for the *Cxcl2-Egfp* reporter were administered *Cn* by orotracheal instillation (10^4^ yeasts cells/mouse, H99 strain) and cells from the lungs of infected and naïve control mice were harvested 9 hrs post-instillation. CD45^+^ cells from lung homogenates were isolated using MACS beads. Cells from three mice per group were pooled for subsequent analysis. Intra-vascular cells were not depleted prior to cell isolation. Because of this, the dataset contains both extra- and intra-vascular lung immune cells. scRNA-seq was then performed on CD45^+^ cell samples with the 10X Genomics Chromium platform. Detailed scRNA-seq library preparation methods were previously described (15).

### Reference-Based Annotation of Immune Cell Subsets

Cell Ranger v3.0.1 (10X Genomics) was used to process raw sequencing files into fastq format as previously described (15). Briefly, reads were aligned with a modified mouse mm10 transcriptome containing the *Egfp* transgene along with all protein coding and long non-coding RNA genes. CellRanger was used to generate a matrix file with expression counts for each sample, with genes as rows and cell Unique Molecular Identifier (UMI) as columns. We obtained 5,635 median UMI counts per cell, 1771 median genes per cell, and 82,314 mead reads per cell.

Seurat v3.1.0 (16) was used to calculate the number of expressed genes, counts per cell, and the percentage of mitochondrial genes as previously described (15). The following criteria were used to filter cells: total number of genes between 200 and 20,000; number of counts between 500 and 75,000; mitochondrial gene frequency <10%. A total of 4,586 cells from naïve and 5,694 cells from infected samples were used for downstream analysis. The SCTransform method (17) was used to perform normalization and variance stabilization of expression counts using regularized binomial regression, with regression on percent mitochondrial genes per cell (15). For cell-type identification, integration between samples was performed using the anchor-based canonical correlation analysis (CCA) method. PCA was then performed, and the top 50 principal components were selected for Uniform Manifold Approximation and Projection (UMAP) for two-dimensional projection. Calculation of k-nearest neighbors and cluster identification were performed.

SingleR (18) was used to perform automated reference-based annotation using curated bulk RNA-seq data from ImmGen for major immune populations as previously described (15). We also examined expression of canonical cell-type specific marker genes as well as unbiased cluster-specific markers for each population to confirm our annotation of major immune cell populations. This allowed us to confidently identify neutrophils within our dataset by confirming neutrophil canonical markers (*S100a8*, *Ly6g*). Detailed results of reference-based annotation can be found in Xu-Vanpala et al. (15).

### Cluster Analysis of Neutrophils

Cells annotated as neutrophils in the scRNA-seq dataset were selected for further analysis using Seurat v3.1.0 (16). Normalization and variance stabilization with SCTransform (17) and PCA were performed, and the top 40 principal components were selected for UMAP visualization. Calculation of k-nearest neighbors and cluster identification was performed. Hierarchical clustering of the neutrophil subsets was used to annotate major branches (I, II, III) and subclusters (*i.e.* IIa, IIb). Numbering of the clusters was based on relative size of each subpopulation. Cluster-specific expression markers were identified, specifically focusing on upregulated genes.

### Pathway Enrichment Analysis

Pathway enrichment analysis was performed on cluster-specific markers using *ReactomePA* (19). Among the resulting enriched pathways with adjusted p-value<0.05, top hits were selected and plotted in a heatmap as -log(adjusted p-value) to illustrate shared enriched pathways between clusters.

### Gene Regulatory Network Analysis

*SCENIC* (single-cell regulatory network inference and clustering) (20) was used to identify transcription factors predicted to regulate neutrophil heterogeneity. Default parameters were used for the *SCENIC* workflow in R and the normalized single-cell gene expression matrix from Seurat was used as input. Co-expression analysis was performed with *GENIE3*. For visualization, we calculated the average regulon activity (AUC) scores for each neutrophil cluster and selected the top regulons to plot as a heatmap using *pheatmap*.

### Ligand-Receptor Interaction Analysis

NicheNet (*nichnetr*) (21) was used to identify predicted ligand-receptor interactions between neutrophil subsets of interest (Ox-PMN, Cyt-PMN) and myeloid cell types of interest (AMs, DCs) using default parameters for the workflow in R. AMs and DCs were separately selected as the “receiver cell types” for independent analyses and the condition of interest selected was “post-*Cn* instillation” compared to “naïve”. Neutrophils were selected as the “sender cell type” and the set of potential ligands was defined as the combined list of Ox-PMN (IIa) and Cyt-PMN (IIb) marker genes. Top predicted ligand-receptor interactions were visualized using the *circlize* R package (22) as a circos plot in which link transparency and width reflected the regulatory potential of a given ligand-target interaction.

## Results of Single Cell RNA-Seq Analysis

### Acute *Cryptococcus neoformans* Pulmonary Infection Induces Neutrophil Subsets With Distinct Transcriptional Profiles

**Figure 1:**
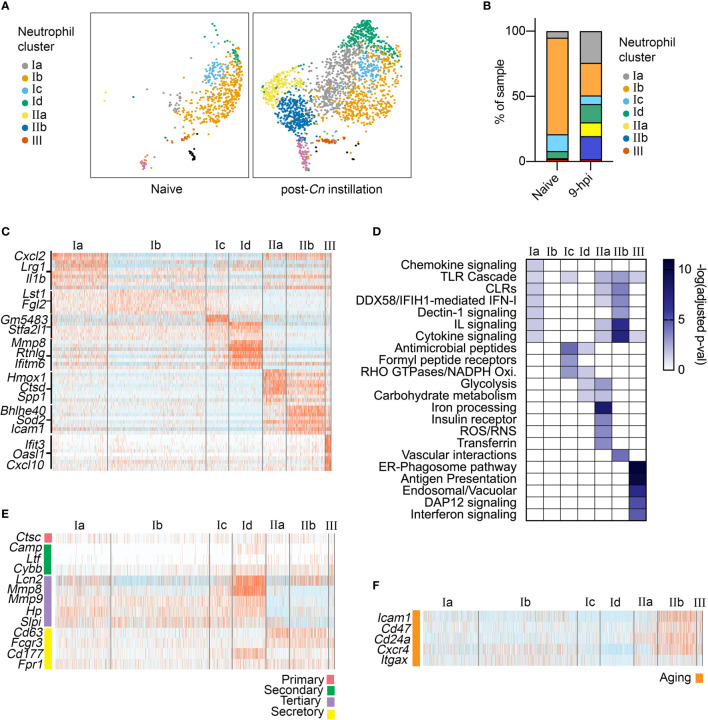
Single-cell RNA-seq analysis of neutrophil heterogeneity following acute pulmonary *C neoformans* infection. **(A)** UMAP projection of neutrophils (611 neutrophils in naïve condition, 2,139 neutrophils at 9-hpi), colored by clusters and separated by condition. Samples were pooled from three mice per group for analysis from a single experiment. **(B)** Frequency of each neutrophil subset, comparing naïve and infected samples. **(C)** Heatmaps showing expression of subset-enriched markers. **(D)** Heatmap of -log (Adjusted p-value) for Reactome pathway enrichment in different neutrophil subsets. **(E, F)** Heatmaps showing expression of select neutrophil granule genes **(E)**, markers of aged neutrophils **(F)**.

To characterize the immune response to acute *Cn* infection, we performed analysis of a previously published scRNA-seq dataset consisting of lung immune cells from naïve and *Cn*-infected mice. Specifically, CD45^+^ immune cells were isolated from the lungs of mice 9-hr post infection (hpi) with *Cn* (10^4^ yeasts cells/mouse, H99 strain, administered by orotracheal instillation) and compared to cells from naïve controls, including both extra- and intravascular lung immune cells (15). Because we sought to understand early host responses, we selected the 9-hpi timepoint, at which the level of a neutrophil chemoattractant CXCL2 has already increased in bronchoalveolar lavage fluid and neutrophils start to infiltrate in the lung (15). Reference-based annotation with *SingleR* (18) was used for initial classification of immune cell types in the dataset, including neutrophils. In the infected mice, we observed a dramatic increase in the relative frequency of neutrophils among CD45^+^ immune cells in our dataset (GEO: GSE146233) (15). Among neutrophils, we identified three major neutrophil subsets, termed clusters I-III (**Figures 1A, B**). While clusters I and III were present in both naïve and infected conditions, cluster II only emerged following *Cn*-exposure (**Figures 1A, B**).

To identify potential functions of neutrophil subsets, we performed pathway enrichment analysis on cluster-specific markers (**Figures 1C, D**). Overall, total cluster I neutrophils did not exhibit very distinct marker expression, although subclusters Ic and Id showed enrichment of genes encoding antimicrobial peptide and NADPH oxidase pathways (*S100a8/9, Lyz2, Pglyrp1*). In contrast, cluster III neutrophils showed strong enrichment of Interferon signaling pathway genes (*Oasl1, Isg15, Irf9*), similar to recently described interferon stimulated genes (ISG) expressing blood neutrophil subset (23). While cluster I and III neutrophils were present in both naive and post-*Cn* instillation conditions, cluster II neutrophils were specific to the *Cn-*stimulated condition and thus are of greater interest in this study. Among cluster II neutrophils, we identified two distinct subsets. Both subclusters IIa and IIb were enriched in PRR signaling pathway genes, specifically Toll-like receptor (TLR) cascades and C-type lectin receptors (CLRs), particularly Dectin-1 signaling (**Figure 1D**). However, IIb cells showed the greatest enrichment in interleukin and cytokine signaling pathway genes (*Il1a, Csf1, Tnf*), while IIa cells showed enrichment in iron processing (*Hmox1, Hmox2*), ROS/RNS (*Atp6v1e1*), and glycolysis (*Pfkl, Gapdh*) pathway genes. Based on these markers, we named cluster IIa as oxidative-signature neutrophils (Ox-PMN) and cluster IIb as cytokine-signature neutrophils (Cyt-PMN). We hypothesize that Ox-PMN and Cyt-PMN may represent distinct neutrophil activation states triggered by acute *Cn* exposure.

To investigate whether specific neutrophil subsets reflect different stages of neutrophil maturation, we examined expression of neutrophil ageing-related markers (24), as well as granule and secretory vesicle components, which are typically expressed sequentially during neutrophil development (25). We observed enrichment of tertiary granule factors (*e.g.*, *Mmp8*) in subcluster Id neutrophils (**Figure 1E**) and markers of ageing (*e.g.*, *Icam1*) in subcluster IIb (**Figure 1F**). Subcluster Id expression of tertiary granule factors suggests these neutrophils are transitioning from the metamyelocyte stage to the band stage, and thus may be more immature neutrophils (25). In contrast, IIb (Cyt-PMN) showed elevation of genes associated with neutrophil ageing (*Cxcr4* and *Icam1*). In addition, Cyt-PMNs also showed enrichment in Bcl2 family genes (*Bcl2a1a, Bcl2a1d, Bcl2a1b*) encoding anti-apoptotic proteins and thus may facilitate or respond to neutrophil ageing.

In summary, based on single-cell transcriptional profiling, we hypothesize that acute *Cn* exposure induces two distinct neutrophil activation states: Ox-PMN (subcluster IIa) and Cyt-PMN (subcluster IIb) with distinct gene expression profiles and functions. In addition, we found the Cyt-PMN are also enriched in markers of neutrophil ageing, suggesting that Cyt-PMN may be longer-lived than other neutrophil subsets profiled.

### Distinct Transcription Factors Are Predicted to Regulate Ox-PMN and Cyt-PMN Subsets

**Figure 2:**
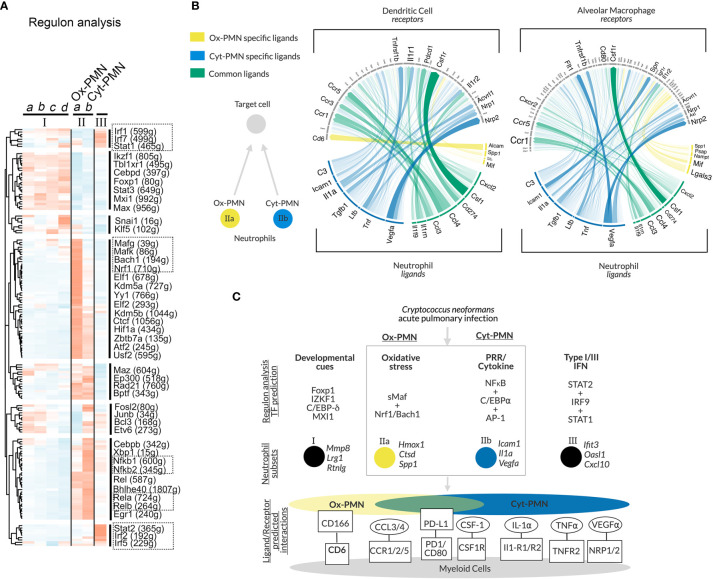
Gene regulatory network analysis of neutrophil subsets and predicted cell-cell communication – **(A)** Heatmap of regulon activity across neutrophil subsets with hierarchical clustering of regulons displayed in dendrogram on left-hand side. Selected TFs of interest are highlighted by boxes with dotted lines. The number of predicted genes targeted by the TFs are indicated with parentheses. **(B) **Circos plot showing arrows between IIa-specific (yellow), IIb-specific (blue) or IIa/b (green) ligands and their target receptors on DCs (left panel) or AMs (right panel). Significance of potential interaction is indicated by the opacity and thickness of the connecting arrow. **(C)** Hypothesized model of neutrophil subset regulation during acute pulmonary *Cn* infection and predicted ligand/receptor interactions with other immune cells.

To identify transcription factors (TFs) that may regulate neutrophil heterogeneity, we used single-cell regulatory network inference and clustering (SCENIC) (20). The method evaluates TFs and *cis*-regulatory sequences to predict biological states of cells. This approach involved three steps: (A) identifying groups of genes that are co-expressed with TFs (“modules”), (B) identifying predicted TF binding sites near co-expressed genes (“regulons”) using motif analysis of the mouse reference genome, and (C) calculating predicted activity of candidate TF regulons across cell subsets (“regulon activity”). The majority of significant TF regulons, which were identified by SCENIC, showed increased activity particularly in cluster II neutrophils. However, IIa and IIb neutrophils (Ox-PMN and Cyt-PMN, respectively) exhibited distinct patterns in predicted activity (**Figure 2A**).

Cyt-PMN-active regulons included NFκB family TFs (encoded by *Nfkb1, Nfkb2, Rel, Relb, Rela*), which mediate neutrophil response to cytokines and PRR signaling. In contrast, Ox-PMN active regulons included small Maf (sMaf) transcription factors (encoded by *Mafk, Mafg*), as well as other TFs in the CNC and Bach families (encoded by *Bach1, Nrf1*), which form heterodimers with sMaf TFs (26). sMaf heterodimerizes with CNC or Bach and mediates cellular responses to oxidative stress (26, 27), although the function of the sMaf heterodimers remains largely uncharacterized in neutrophil biology. Based on this analysis, we hypothesize that Cyt-PMN (IIb) may be regulated by NFκB TFs, while Ox-PMN (IIa) appears to be under control of sMaf, CNC, and Bach TFs.

### Ligand-Receptor Analysis Identifies Potential Neutrophil Interactions With DCs and AMs

To identify potential cell-cell communication between neutrophil subsets (Ox-PMN and Cyt-PMN) and other immune cells during *Cn* infection, we used “NicheNet” to predict ligand-receptor interactions (21). Specifically, this approach leverages prior knowledge of ligand-receptor interactions and intracellular signaling pathways to predict which ligand-receptor pairs may regulate gene expression in target cells. Our interest was in neutrophil interactions with alveolar macrophages (AMs) or dendritic cells (DCs), because these cell types are involved in modulating the immune response during the early stages of infection.

We found that Ox-PMN (IIa) express only a few unique ligands that are predicted to be detected by AMs and DCs (**Figure 2B**). Among these, the strongest potential interaction was between Alcam (Activated leukocyte adhesion molecule) on the Ox-PMN side and CD6 on the DC side. This predicted mechanism for neutrophil-DC communication has not been previously studied to our knowledge. In contrast, Cyt-PMN (IIb) expressed multiple genes encoding ligands with strong predicted interactions with both DCs and AMs. These suggested cell-cell communications *via* IL-1α and IL-1R1/IL1-R2, TNFα and TNFR2, and VEGFα and NRP1/NRP2. While the role of VEGFα in *Cn* infection is less understood, both IL-1α and TNFα are important drivers of anti-fungal response to *Cn* (12). Among ligands expressed by both Cyt-PMN and Ox-PMN neutrophils, CCL3 and CCL4 are notable chemo-attractants for immune cells expressing CCR5, which is important for host immune response to *Cn* (28). Other ligand-receptor pairs identified include PD-L1 (*Cd274*) and PD-1 (*Pdcd1*), as well as CSF1 (*Csf1*) and CSF1R (*Csf1r*). The role of these ligand-receptor pairs between neutrophils and DCs (or AMs) merits further investigation.

In summary, our analysis suggests that both Cyt-PMN and Ox-PMN neutrophils express common ligands (CCL3, CCL4), which are detected by DCs and AMs through CCR5 and CCR1, respectively. However, inflammatory cytokine expression, including IL-1α and TNFα, may be specific to Cyt-PMN subset. Furthermore, Cyt-PMNs also appear to specifically express complement C3, critical for complement-mediated phagocytosis of *Cn* by DCs and other phagocytes. Thus, the Cyt-PMN subset may promote phagocytosis and anti-fungal immune responses *via* ligand-receptor interactions with other immune cells.

## Discussion and Hypothesis

In this study, we performed in-depth scRNA-seq analysis of a published dataset and identified multiple neutrophil subsets. Among the subsets, we focused on Ox-PMN (IIa) and Cyt-PMN (IIb), which appear after *Cn* infection and possess distinct gene expression profiles. As summarized in **Figure 2C**, our analysis suggests that Ox-PMN are regulated by sMaf, CNC, and Bach TFs, and these signaling pathways may mediate oxidative stress response and regulate oxidative burst in response to *Cn*. In contrast, the gene expression profile of Cyt-PMN is highly enriched with genes encoding NFκB signaling molecules and pro-inflammatory cytokines, IL-1α and TNFα. Based on ligand-receptor analysis, these cytokines are predicted to mediate cross-talk of Cyt-PMN with DCs and AMs. In contrast, fewer unique interactions were found between Ox-PMN-specific ligands and receptor on DCs and AMs. With our data, we hypothesize that, during acute pulmonary *Cn* infection, there is a division of labor, in which activated neutrophils become either ROS-producing Ox-PMN or cytokine-producing Cyt-PMN.

As reflected in distinct gene expression patterns, we also hypothesize that neutrophils subsets have distinct spatial localization in the lung. For example, cluster II neutrophils including Ox-PMN and Cyt-PMN are infection-specific subsets; thus, we expect that they are located in the lung parenchyma, where the subsets are exposed to *Cn* and exert subsequent immune responses. In contrast, the cluster Ib neutrophil subset showed a quiescent gene expression phenotype and were mainly identified in naïve mice. Thus, we expect the Ib subset to be mainly found in the lung vasculature. It is possible that cluster III neutrophils also localized in the lung vasculature because their IFN-signature gene expression profile is similar to a subset of steady-state neutrophils in circulation (23).

We predict that Cyt-PMN may consist of aged neutrophils based on their expression of markers of neutrophil ageing. It is also possible that Cyt-PMN further facilitate enhanced cytokine production over an extended period of time. In contrast, Ox-PMNs may reflect neutrophils which are in direct contact with or have phagocytosed *Cn* based on the pattern of highly expressed genes related to oxidative stress responses. Ox-PMNs may also be poised to undergo NETosis, triggered by ROS. To test these hypotheses, subset-specific markers and ROS indicators can be used to identify Cyt-PMN and Ox-PMN for analysis of location, maturity, interaction with *Cn*, and NETosis.

A previous study, using a *Staphylococcus aureus* infection model, identified two distinct neutrophil subsets, described as PMN-I and PMN-II with their dichotomous gene expression patterns characterized by highly expressed IL-12 and IL-10, respectively (29). However, we did not find a similar dichotomous pattern between Cyt-PMN and Ox-PMN. Instead, Ox-PMN gene expression reflects an activation state likely triggered by ROS production or exposure to ROS, while that of Cyt-PMN reflect PRR and cytokine receptor signaling to mediate NFκB-driven *Il1a* and *Tnf* production.

Although neutrophils are known to have anti-fungal functions, neutrophil depletion leads to a paradoxical increase in survival of animals in pulmonary cryptococcosis (11–13). These paradoxical results may reflect the delicate balance of heterogeneous subsets in neutrophils: Some subsets may be host-protective, and others may be host-detrimental. Thus, specific targeting of Ox-PMN and Cyt-PMN subsets may help clarify neutrophil functions during pulmonary *Cn* infection. This could be accomplished by either (A) targeting upstream transcription factors and signaling pathways predicted to regulate Ox-PMN and Cyt-PMN identity, (B) using subset-specific markers for targeted depletion studies, or (C) targeting predicted functions such as ROS-production or cytokine production in a neutrophil-specific manner. Furthermore, understanding and testing the role of cell-cell interactions between Cyt-PMNs and other myeloid cells could also clarify neutrophil function in pulmonary *Cn* infection. Beyond known pro-inflammatory cytokines such as IL-1α (*Il1a*) and TNFα (*Tnf*), we also found that both Ox-PMN and Cyt-PMN are predicted to interact with other myeloid cells through expression of PD-L1 (*Cd274*), VEGFα (*Vegfa*), and CSF1 (*Csf1*). These factors are relatively uncharacterized in neutrophils, and elucidating their roles is a novel area for investigation in neutrophil biology more broadly.

In this study, we developed a hypothesis that acute pulmonary *Cn* infection leads to distinct neutrophil activation states, which is exampled by a division of labor between ROS-producing Ox-PMN and cytokine-producing Cyt-PMN. In addition to proposing specific testable questions for investigation of neutrophils in *Cn* infection, it will also be important to understand whether this form of neutrophil heterogeneity is broadly relevant in fungal infections. In summary, our in-depth analysis of single-cell RNA sequencing of lung neutrophils provides both a detailed reference and theoretical model to guide new studies of neutrophil function in *Cn* infection.

## Limitations of study

In this Hypothesis and Theory article, we analyzed single-cell transcriptional heterogeneity of neutrophils in acute *Cn* pulmonary infection and developed a theoretical framework for future investigation of neutrophil function during *Cn* infection. We acknowledge that we used three pooled mice per group for analysis without distinguishing intra-vascular from extra-vascular neutrophils in the lung. Our findings are limited to the 9-hpi timepoint. Future studies using multiple timepoints during infection, as well as validation of neutrophil phenotypes and functions, would be valuable to characterize the full dynamics of immune cell transcriptional heterogeneity in pulmonary *Cn* infection.

## Data Availability Statement

The datasets presented in this study can be found in online repositories. The names of the repository/repositories and accession number(s) can be found below: https://www.ncbi.nlm.nih.gov/, GSE146233.

## Ethics Statement

The animal study was reviewed and approved by Duke University IACUC.

## Author Contributions

MED performed data analysis and created figures for the manuscript under the guidance of MLS. MED, EYR, SX-V, and MLS drafted, edited and revised the manuscript. All authors contributed to the article and approved the submitted version.

## Funding

This work was supported in part by the NIH to MS (R01-AI088100), MD (F30-AI140497, T32-GM007171), and ER (T32-AI052077).

## Conflict of Interest

The authors declare that the research was conducted in the absence of any commercial or financial relationships that could be construed as a potential conflict of interest.
